# Cardiac repair in a mouse model of acute myocardial infarction with trophoblast stem cells

**DOI:** 10.1038/srep44376

**Published:** 2017-03-15

**Authors:** Guannan Li, Jianzhou Chen, Xinlin Zhang, Guixin He, Wei Tan, Han Wu, Ran Li, Yuhan Chen, Rong Gu, Jun Xie, Biao Xu

**Affiliations:** 1 Department of Cardiology, Nanjing Drum Tower Hospital, the Affiliated Hospital of Nanjing University Medical School, Nanjing, Jiangsu, 210008, China; 2 Department of Cardiology, the First Affiliated Hospital of Guangxi University of Chinese Medicine, Nanning, Guangxi, 530023, China

## Abstract

Various stem cells have been explored for the purpose of cardiac repair. However, any individual stem cell population has not been considered as the ideal source. Recently, trophoblast stem cells (TSCs), a newly described stem cell type, have demonstrated extensive plasticity. The present study evaluated the therapeutic effect of TSCs transplantation for heart regeneration in a mouse model of myocardial infarction (MI) and made a direct comparison with the most commonly used mesenchymal stem cells (MSCs). Transplantation of TSCs and MSCs led to a remarkably improved cardiac function in contrast with the PBS control, but only the TSCs exhibited the potential of differentiation into cardiomyocytes *in vivo*. In addition, a significantly high proliferation level of both transplanted stem cells and resident cardiomyocytes was observed in the TSCs group. These findings primary revealed the therapeutic potential of TSCs in transplantation therapy for MI.

The massive cardiomyocytes loss following myocardial infarction (MI) predisposes to progressive left ventricular remodeling, heart failure, and causes substantial mortality and morbidity^1^,^2^,^3^. Current treatment approaches aim to prevent further injury and block the maladaptive pathways^3^,^4^, but not seek to compensate for the lost myocardium, which is critical owing to the limited regenerative potential of adult cardiomyocytes^5^,^6^. Therefore, stem cell therapy has emerged as a novel strategy for MI treatment^7^,^8^.

Various types of stem cells have been proposed for cardiac cell therapy^8^,^9^, such as skeletal myoblasts, mesenchymal stem cells (MSCs), hematopoietic stem cells (HSCs), and embryonic stem cells (ESCs). Although several publications describe that stem cell therapy facilitates the recovery of cardiac function post-MI^7^,^10^, the individual populations of stem cells have limitations that challenge their common usage^8^,^9^,^11^, including poor cardiomyocytes differentiation, severe arrhythmia, and tumor formation. Thus, “which is the optimal stem cell population” remains an open question in this field.

Trophoblast stem cells (TSCs) are a population of stem cells originating from a single layer of blastocysts when the earliest cell differentiation occurs^12^. During embryonic development, TSCs contribute to the formation of the placenta, which ensures the exchange of nutrients and oxygen between the mother and the fetus^13^. Accumulating evidence supports that TSCs have extensive plasticity and are capable of differentiating into a variety of cell morphologies, such as primary trophoblast giant cells, spongiotrophoblast, glycogen cells, and pluripotent stem cells^14^,^15^,^16^. Strikingly, Chaudhry *et al*.^17^ recently showed that fetal cells selectively homed to injured maternal hearts and differentiated into cardiomyocytes, endothelial cells, and smooth muscle cells, and about 40% of the fetal cells in the maternal heart expressed caudal-related homeobox2 (Cdx2), a typical marker of TSCs. Therefore, TSCs might be promising for myocardium regeneration owing to their excellent homing capacity and differentiation into cardiac lineage cells. Here, we firstly evaluated the feasibility and efficacy of TSCs transplantation for heart regeneration in a mouse model of MI and determined whether TSCs were superior to MSCs.

## Results

### Characterization of TSCs

**Figure 1:**
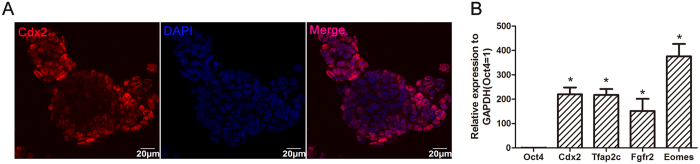
Characterization of TSCs and MSCs. (**A**) TSCs expressed the specific marker, Cdx2, by immunofluorescent staining. (**B**) TSCs expressed markers of Cdx2, Tfap2c, Fgfr2, and Eomes by RT-PCR, but did not express Oct4 (*P < 0.01). Data are represented as mean ± SD.

TSC lines were isolated from day 3.5 blastocysts of pregnant GFP-transgenic mice^14^. Passage 2 cells were cultured in fibroblast-conditioned medium (Supplementary Fig. S1). The expression of the TSCs-specific transcription factor, Cdx2^18^, was detected in the established TSCs by immunofluorescent staining (Fig. 1A). Quantitative real-time polymerase chain reaction (qRT-PCR) data also showed that TSCs marker genes^15^, including Cdx2, Tfap2c, Fgfr2, and Eomes, were highly expressed in cultured cells, whereas Oct4, the ESCs marker was silenced in the cells (Fig. 1B).

MSCs were isolated from the bone marrow of GFP-transgenic mice, and cultured as described previously^19^. Immunofluorescent staining demonstrated that MSCs were positive for CD90.2 and CD105, and negative for CD31, a marker of endothelial progenitor cells (EPCs), as well as CD45 and CD117, markers of HSCs (Supplementary Fig. S2A). Flow cytometry analysis further confirmed the characteristic immunophenotype of MSCs (Supplementary Fig. S2B).

### TSCs transplantation improved cardiac function after MI

**Figure 2:**
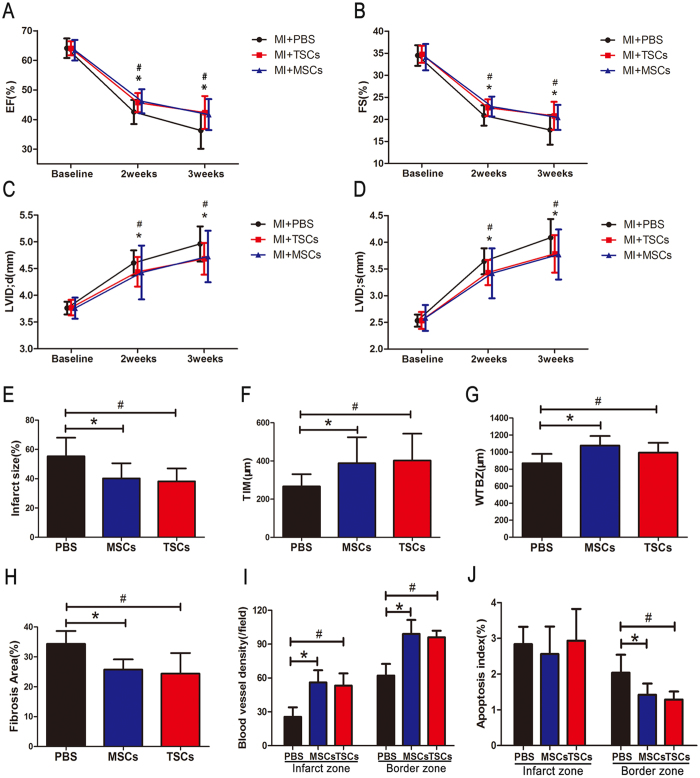
Cardiac function and remodeling after cell transplantation. (**A**–**D**) Sequential echocardiographical assessments of the infarcted mice at baseline, 2 weeks, and 3 weeks showed an increase in EF and FS, as well as, a decrease in LVIDd and LVIDs in TSCs- or MSCs- treated animals as compared to the PBS group. (**E**) Infarct size expressed as a percentage of left ventricular area in the cell therapy groups was significantly smaller than the PBS treated group. (**F**,**G**) TIM and WTBZ in cell-treated hearts were substantially higher than the PBS-treated hearts. (**H**) Quantification of interstitial fibrosis in the border zone of the three groups revealed that the cell therapy decreased the fibrosis of hearts after MI. (**I**) Quantification of CD31^+^ capillaries suggested that the density of vessels in the cell-treated groups were significantly higher than in the PBS-treated group both in the infarct and border zones. (**J**) Quantification of Tunel^+^ cells showed that the apoptotic cells in the cell-engrafted groups were significantly lower than in the PBS-treated group at the border zone, but no difference was seen in the infarct zone among the three groups. n = 8 for each group. *P < 0.05, MSCs-treated group vs. PBS-treated group; ^#^P < 0.05, TSCs-treated group vs. PBS-treated group. Data are depicted as mean ± SD.

After the cell lines had been established *in vitro* successfully, vehicles (PBS) or cells (5 × 10^5^ MSCs or 5 × 10^5^ TSCs) were administered into the hearts undergoing MI through intramyocardial injection. The cardiac function was evaluated by echocardiography at baseline, as well as 2 and 3 weeks after cell transplantation. We observed significant improvement in the left ventricular ejection fraction (EF) and fractional shortening (FS) and a significant decrease in the end-diastolic left ventricular inner diameter (LVID;d) and end-systolic left ventricular inner diameter (LVID;s) in mice (Fig. 2A–D). However, no significant difference was detected between the TSCs and MSCs groups with respect to all the echocardiography parameters.

Three weeks after cell transplantation, the mice were sacrificed, and histological analysis was performed. The infarct size and wall thickness were determined by hematoxylin and eosin (HE) staining. The infarct size was significantly smaller in hearts receiving TSCs or MSCs compared to the PBS group (Fig. 2E and Supplementary Fig. S3). The thickness of infarcted myocardium (TIM) (Fig. 2F) and a wall thickness of border zone (WTBZ) (Fig. 2G) were also significantly greater in cell-treated hearts than in PBS only. Nevertheless, no significant difference was found between the two types of stem cells.

Tumorigenesis is a primary risk associated with stem cell therapy. Here, we detected tumor tissue in the hearts, livers, and kidneys of two stem cells-treated mice, on HE-stained sections. Interestingly, no tumor formation was observed in the above major organs of mice, with TSCs and MSCs, 3 weeks after injection (Supplementary Fig. S4).

Together, these results showed that transplantation of TSCs or MSCs improved the cardiac function after MI in mice. However, no statistical differences were noted in cardiac function and LV morphometry, when comparing the TSCs and MSCs groups.

### TSCs transplantation reduced fibrosis, cell apoptosis, and enhanced angiogenesis after MI

We next evaluated the effect of stem cell transplantation on the remodeling of injured hearts. Masson trichrome staining was performed for interstitial fibrosis in the border zone. At 3 weeks after MI, collagen content within the border zone was reduced in either of the stem cell-treated groups in contrast to the PBS control group (Fig. 2H and Supplementary Fig. S5A).

We measured the capillaries in the infarct zone and the border zone by immunohistochemistry staining of CD31 at 3 weeks. the capillary density was significantly higher in the groups that received TSCs or MSCs than in the control group both in the infarct and border zones (Fig. 2I and Supplementary Fig. S5B).

Cell apoptosis was quantified by TUNEL assay. In the border zone, the percentage of TUNEL-positive cells was markedly reduced in the cell-treated hearts compared to the hearts that received PBS alone. However, the cell apoptosis in the infarct zone of the three groups did not achieve any statistical significance (Fig. 2J and Supplementary Fig. S5C).

### TSCs showed an enhanced retention than MSCs after transplantation into injured hearts

**Figure 3:**
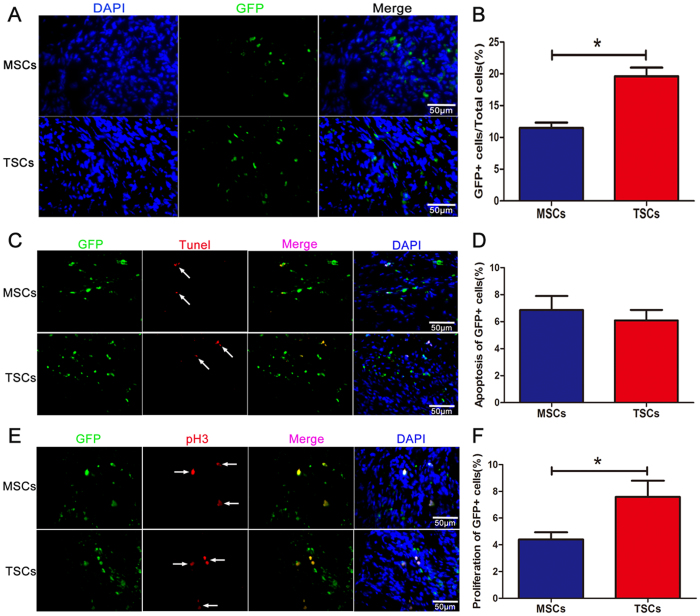
Retention of MSCs and TSCs after transplantation. (**A**) The retention of injected cells was detected by the percentage of GFP^+^ in all the cells in every field under fluorescent microscopy, and nuclei were counterstained with DAPI. (**B**) Quantification of GFP^+^ cells to total cells showed that the retention of TSCs was higher than the MSCs (*P < 0.05) (Table S1). (**C**) The co-expression of GFP and Tunel indicated the apoptosis of implanted cells *in vivo*. (**D**) Quantification of co-expression of Tunel and GFP cells showed no difference between TSCs and MSCs in apoptosis after transplantation (Table S3). (**E**) The co-expression of GFP and pH3 indicated the proliferation of implanted cells *in vivo*. (**F**) Quantification of co-expression of pH3 and GFP cells showed that TSCs exhibited more proliferation than MSCs *in vivo* (*P < 0.05) (Table S2). n = 6 for each group. Data are depicted as mean ± SD.

Transplanted stem cells were detected in the infarct and border zone 3 weeks after treatment. Since the transplanted cells had been isolated from GFP-transgenic mice, GFP-positive cells were detected in the infarct and border zone by fluorescent microscopy (Fig. 3A). We found that the proportion of cells expressing GFP was higher in the hearts transplanted with TSCs (19.60 ± 1.25% of all cells) than those with MSCs (11.49 ± 0.76% of all cells) (Fig. 3B and Supplementary Table S1).

To investigate the fate of the transplanted cells under pathological conditions after injection, we performed the TUNEL assay to assess apoptosis and the mitotic marker of phosphorylated Histone-H3 (pH3) staining for proliferation assay (Fig. 3C and E). The number of proliferative stem cells was significantly higher in TSCs-treated hearts (7.75 ± 1.17%) than those treated with MSCs (4.40 ± 0.49%) (Fig. 3F and Supplementary Table S2), whereas the apoptosis of transplanted cells was similar between the two groups (6.09 ± 0.72% in TSCs and 6.86 ± 0.95% in MSCs) (Fig. 3D and Supplementary Table S3).

These observations showed that TSCs had a higher retention compared to MSCs after injection *in vivo*, which may be correlated to higher proliferation.

### Transdifferentiation of TSCs after transplantation

**Figure 4:**
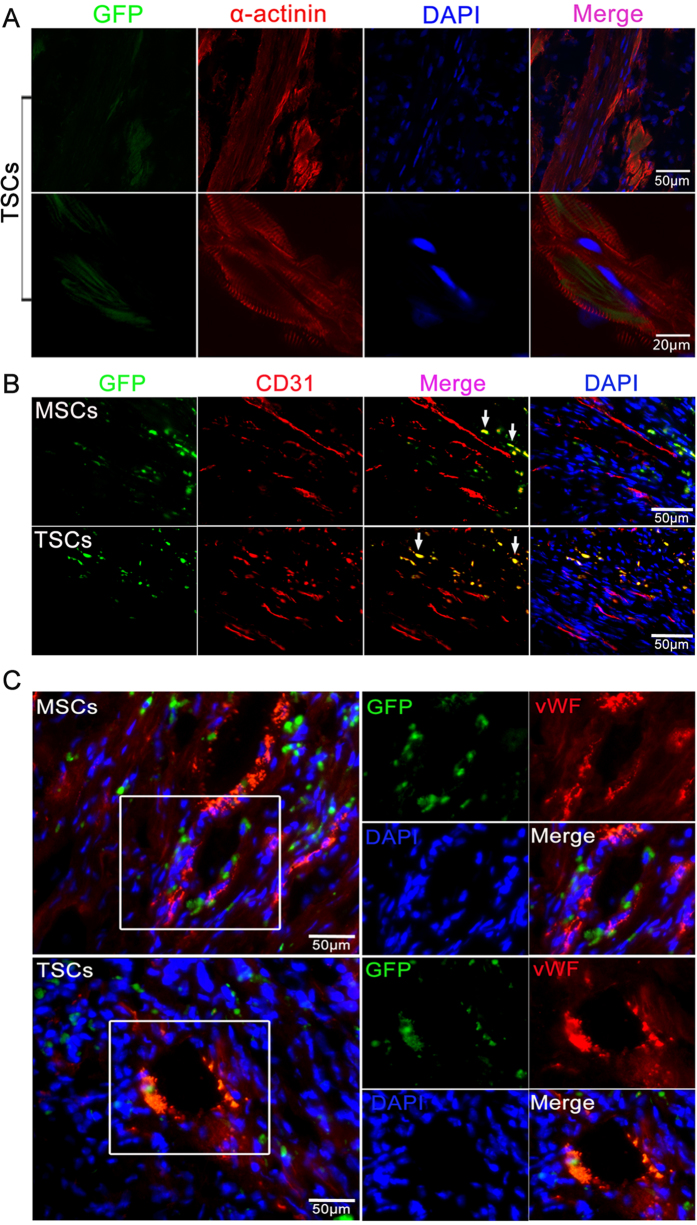
Transdifferentiation of TSCs and MSCs *in vivo*. (**A**) Confocal imaging of the TSCs-engrafted hearts and fluorescent immunostaining for α-actinin demonstrated evidence for cardiomyocytes differentiation; nuclei were counterstained with DAPI. (**B,C**) The sections displayed in **B** and **C** showed that MSCs and TSCs gave rise to endothelial-like cells by CD31-staining and participated in the formation of the vasculature as assessed by vWF staining *in vivo*. The arrows in **B** identifies co-staining of GFP and CD31. The boxed region in **C** is shown at higher magnification in the right panel. n = 6 for each group.

A previous study showed that Cdx2-positive TSCs could differentiate into beating cardiomyocytes when cultured with feeder cardiomyocytes *in vitro*^17^. Whether TSCs could exhibit the same ability of differentiation *in vivo* needs to be elucidated. Thus, we observed the colocalization of GFP and the cardiomyocyte-specific marker, α-actinin, by immunofluorescence. We found that GFP colocalized with α-actinin in hearts receiving TSCs, but not MSCs, providing evidence that TSCs committed to cardiomyocytic lineage (Fig. 4A). However, the number of TSCs committed was quite low.

Next, we found that GFP colocalized with the endothelial cell surface marker, CD31, indicating that the transplanted cells gave rise to endothelial-like cells *in vivo* (Fig. 4B). On the other hand, both the TSCs and MSCs seemed to incorporate in the formation of the coronary vasculature as identified by co-staining with a vascular marker (von Willebrand factor, vWF) (Fig. 4C).

### TSCs transplantation exhibited increased cell proliferation than MSCs, especially the cardiomyocytes proliferation

**Figure 5:**
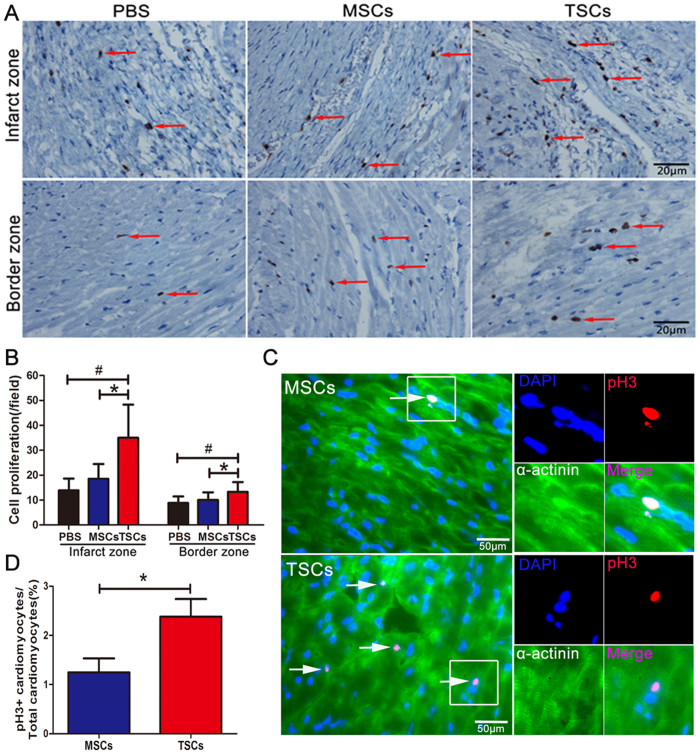
Cell proliferation after cell transplantation. (**A**) Representative Ki67-stained histological sections at 3-weeks after cell transplantation. (**B**) Quantification of Ki67^+^ cells showed that proliferating cells in the TSCs-engrafted groups were significantly higher than the MSCs-treated and PBS-treated groups both in the infarct and border zones; however, no difference was observed between the MSCs-treated group and PBS-treated group (n = 8 for each group). (**C**) Representative pH3-stained sections showed the proliferation of endogenous cardiomyocytes in the border zone at 3 weeks after cell transplantation. The boxed region is shown at higher magnification in the right panel. (**D**) Quantification of pH3^+^ cells showed that the endogenous proliferation cells in the TSCs-treated group were significantly higher than in the MSCs-treated group (n = 6 for each group) (Table S4). *P < 0.05, TSCs-treated group vs. MSCs-treated group; ^#^P* < *0.05, TSCs-treated group vs. PBS-treated group. Data are represented as mean ± SD.

Increased cell proliferation could contribute towards improving the heart function. Therefore, we assessed whether transplantation of stem cells promotes cell proliferation in the MI hearts. Ki-67 expression showed that cell proliferation was markedly increased in the TSCs-treated group compared to the other two groups both at infarct and border zones (Fig. 5A and B). Importantly, the difference between TSCs and MSCs treatments was also significant.

The previous study showed that the transplantation of MSCs stimulated endogenous cardiomyocytes turnover, including endogenous CPCs and resident cardiomyocytes^20^. Here, the immunofluorescence data revealed that the host cardiomyocyte turnover was 2-fold higher in TSCs-treated hearts than MSCs at 3 weeks after therapy, as indicated by the expression of pH3 (1.25 ± 0.26% in MSCs and 2.38 ± 0.33% in TSCs) (Figs. 5C and D, Supplementary Table S4).

### Microarray analysis

**Figure 6:**
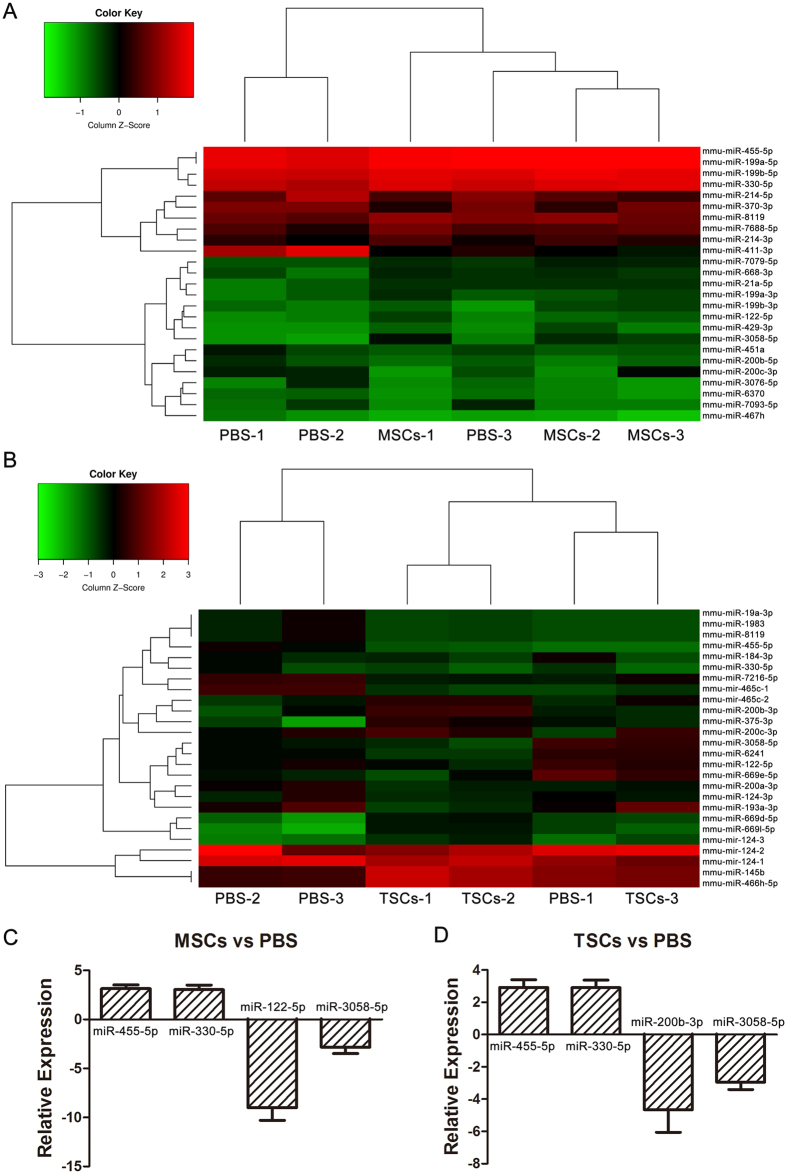
Microarray analysis of the hearts after cell therapy. (**A,B**) Heat map and hierarchical clustering. The heat map shows the result of the two-way hierarchical clustering of miRNAs and samples. Each row represents a miRNA (fold change >2.0) and each column represents a sample (n = 3 for each group). (**A**) Showed the different miRNAs between MSCs and PBS groups. (**B)** Showed the different miRNAs between TSCs and PBS groups. The color scale shown in the top panel illustrates the relative expression level of a miRNA in the certain slide: red color represents a high relative expression level; green color represents a low relative expression level (Table S5). (**C,D**) Validation of miRNA microarray results by RT-PCR (fold change >2.0 and P < 0.05). (**C**) showed the confirmed miRNAs between MSCs and PBS groups (n = 3 for each group). The confirmed miRNAs are provided in Table S6. D showed the confirmed miRNAs between TSCs and PBS groups (n = 3 for each group). Data are depicted as mean ± SD.

We found a higher proliferative capacity in both the transplanted TSCs and the resident cardiomyocytes in TSCs-treated hearts than the MSCs. However, the molecular mechanism of this difference remains unclear. We suspected that miRNAs, which are involved in the regulation of a variety of genes and functional processes^21^, might contribute to the proliferative effect of TSCs. The hearts of the three groups were analyzed by miRNA analysis using Affymetrix GeneChip miRNA 4.0 Array. The fold-change filtering (fold change > 2.0) of the differentially expressed miRNAs revealed that 15 miRNAs were up-regulated and 10 miRNAs were downregulated in the MSCs-treated samples compared to the PBS-treated samples. Nine miRNAs were upregulated and 17 downregulated in the TSCs-treated samples (Supplementary Table S5). The results of a two-way hierarchical clustering of the miRNAs and the tissue samples are presented in the heat map illustration (Figs. 6A and B). To identify the significantly differentially expressed miRNAs, we set up a fold change of >2.0 and a P*-*value of <0.05 as the threshold for screening. Seven miRNAs in MSCs group and 5 miRNAs in TSCs group were confirmed by qRT-PCR (Supplementary Table S6). Subsequently, we found that miR-200b-3p expression was significantly lower in TSCs-treated hearts compared to the MSCs-treated hearts (Fig. 6C and D). MiR-200b has been reported to suppress cell proliferation in several cell lines^22^,^23^,^24^,^25^. Thus, we propose that miRNA-200b-3p may also be involved in the regulation of the proliferative effect of TSCs.

## Discussion

The present study, for the first time, evaluates the effect of TSCs on myocardium repair following MI in mice by direct comparison between MSCs and placebo. The main findings are as follows: (1) TSCs transplantation attenuates the unfavorable process of left ventricular remodeling and improves the cardiac contract function; (2) TSCs, but not MSCs, exhibit the property of differentiation into cardiomyocytes; (3) TSCs also differentiate into endothelial cells and participate in the formation of vasculature *in vivo*; (4) TSCs display a high potential in stimulating the proliferation of transplanted stem cells and resident cardiomyocytes, potentially through the regulation of miRNA-200b-3p.

The stem cell populations of cell-based therapy for MI can be broadly separated into two categories: pluripotent stem cells and adult stem cells^8^,^9^. Pluripotent stem cells, which include ESCs and induced pluripotent stem cells (iPSCs), are plagued by their pluripotency, which may lead to a tumor risk. On the other hand, the adult stem cells that include skeletal myoblasts, bone marrow-derived stem cells, MSCs, and EPCs, are limited to use due to the poor capacity of differentiation towards cardiomyocytes. TSCs, a new type of stem cells derived from the trophectoderm of blastocysts, have a moderate potential for differentiation between pluripotent stem cells and adult stem cells and have been clearly characterized to commit to cardiomyocytic and vascular lineages *in vitro*^17^, thereby rendering them a potentially promising stem cell population for cardiac repair.

Our *in vivo* study found that some transplanted TSCs colocalized with a cardiomyocyte-specific marker, α-actinin, and vascular specific markers, CD31, and vWF, indicating that TSCs were also able to commit towards new cardiomyocytes and vascular cells *in vivo*. Notably, such a beneficial differentiation is extremely rare. Kara *et al*. demonstrated that a substantial portion of isolated fetal cells differentiated into spontaneously beating cardiomyocytes when co-cultured with neonatal mice cardiomyocytes^17^. The discrepancy between the *in vitro* and *in vivo* data could probably be attributed to the difference of microenvironment in which TSCs grew. The sophisticated environment in ischemic myocardium, including hypoxia, inflammation, and collagen deposition could greatly affect the fate of implanted stem cells^3^. A variety of strategies, including pharmacological treatment, gene modification, biomaterials engineering, and microRNAs delivery, are being developed to enhance the survival and differentiation of stem cells *in vivo*; however, these strategies remain to be optimized, and the effect is yet to be determined^26^,^27^,^28^,^29^.

Despite that the number of cardiomyocytes differentiated from implanted TSCs was low, a significant improvement in the left ventricular contraction function and a decrease in myocardial remodeling was detected following TSCs injection vs. PBS control. These functional benefits might be related to the effect of TSCs on promoting angiogenesis and proliferation, inhibiting collagen deposition, and cell apoptosis. Several studies in recent years have established that stem cell exerted their beneficial effect via cell cross-talk. MiRNAs have been proved as important messenger molecules, participating in a variety of biochemical and cellular activities, including apoptosis, angiogenesis, and proliferation^21^,^30^. Here, microarray analysis and qRT–PCR revealed the up- and down-regulation of miRNAs expression in TSCs or MSCs-treated hearts compared to the PBS group. We observed a similar change between TSCs and MSCs, including miRNA-455-5p, miRNA-330-5p, and miRNA-3058-5p, which may have contribute towards same phenotypes on fibrosis, angiogenesis, and apoptosis.

In addition, TSCs exhibited higher pro-proliferative potential of stem cells and host cells as compared to the MSCs. Microarray analysis and qRT-PCR revealed the difference in miRNA-200b-3p between TSCs and MSCs. Recent reports indicated that miRNA-200b is highly correlated with cell proliferation^22^,^24^,^25^. Especially, in cardiac cell lines, Yao *et al*.^31^ demonstrated that miR-200b overexpression inhibited the cell growth and downregulated the expression of cyclin D1 and myosin heavy chain (MHC) by regulating the expression of GATA-4.

Importantly, the transplantation of ESCs or iPSCs may result in the formation of teratomas according to the previous studies^32^,^33^, and thus, the safety of stem cell therapy in cardiac repair has always been a concern. In the current study, we examined those organs from where the TSCs may travel away, and no tumor was found, which is probably due to the moderate potential compared to ESCs or iPSCs. On the other hand, the time and number of implanted cells were putative influencing factors. The safety of TSCs application may necessitate an in-depth analysis in future.

Despite these encouraging results and the potential application of the TSCs, there are also limitations in this study. First, the differentiation of TSCs in an *in vivo* environment requires being further enhanced and liberated. Second, the possible paracrine effect of TSCs warrants clear investigation. Lastly, the isolation and culture of TSCs in human remains a challenge, and thus, the clinical application of TSCs seems like a long way.

## Methods

### Animal care and protocol

The investigations were performed in accordance with the Guide for the Care and Use of Laboratory Animals published by the US National Institutes of Health (NIH Publication No. 85–23, revised 1996). The protocols were approved by the Ethics Review Board for Animal Studies of Nanjing Drum Tower Hospital.

### Experimental animals

Ten-week-old male and female GFP-transgenic C57BL/6 mice were used to prepare the TSCs and MSCs. Ten-week-old C57BL/6 wild-type (WT) male mice were used as the model of myocardial infarction in the present study. All mice were obtained from the Model Animal Research Center of Nanjing University.

### Preparation of TSCs and MSCs

TSCs were derived and maintained as described previously^14^. Briefly, day 3.5 blastocysts were flushed from the uterus of C57BL/6-gfp pregnant mice. The blastocysts were then transferred onto mitomycin C (MMC)-treated ICR mouse embryonic fibroblasts (MEFs) with TSCs medium comprising of RPMI1640 (Gibco, Invitrogen), 20% fetal bovine serum (FBS, Hyclone, Logan), 1 mM sodium pyruvate (Sigma), 100 μM β-mercaptoethanol (Millipore, Temecula), 2 mM L-glutamine (Chemicon), and F4H (25 ng/mL fibroblast growth factor 4 (FGF4), Invitrogen and 1.0 μg/mL heparin, Sigma). After disaggregation at 5–7 days, the culture medium was replaced with 70% FCM (feeder condition medium) + 1.5 × F4H and refreshed every alternate day. At the time of first passage, the medium was changed to 70% FCM + F4H.

The mouse bone marrow-derived MSCs were isolated based on the CFU-F method^19^. The bone marrow cells were seeded at 1–2 × 10^7^ cells/100 mm culture dish. After 3 h, the cells were washed twice with PBS to eliminate the non-adherent cells; the attached cells were cultured for 14–16 days. The attached colonies consisting of spindle-shaped cells were observed under a microscope. The colony-forming attached cells were passaged once. The cells were cultured in α-MEM supplemented with 20% FBS, 2 mM L-glutamine, 55 μM 2-mercaptoethanol, 100 U/mL penicillin, and 100 μg/mL streptomycin.

All the cells were cultured at 37 °C in a humidified atmosphere at 5% CO_2_.

### Mouse model of MI and cell transplantation

MI was induced in 10-week-old male C57BL/6 mice by permanent ligation of the left anterior descending coronary artery (LAD) as described in the previous studies^34^. Briefly, the mice were doped with anesthesia (10% chloral hydrate, 0.3 mL/100 g, i.p.) and maintained under artificial ventilation. The chest cavity was opened, and after careful dissection of the pericardium, LAD was permanently ligated using a 7-0 silk suture. This was followed by injection of either TSCs (5 × 10^5^ cells suspended in 20 μL of PBS, n = 20 animals) or MSCs (5 × 10^5^ cells suspended in 20 μL of PBS, n = 20 animals) at 4 different sites along the infarct border zone with final volume of 5 μL with a 31-gauge syringe (Hamilton) at each site. The cells were suspended in PBS for injection. The vehicle animals (n = 14) received PBS (20 μL) injection in a similar manner. Subsequently, the chest and the overlying skin were closed, allowing the animals to recover under aseptic precautions and analgesic medication (Buprenorphine, 0.1 mg/kg s.c.) to reduce the post-operative pain.

### MicroRNA microarray assay

Total RNA was isolated from frozen tissues using the TRIzol reagent (Life Technologies, USA), according to the manufacturer’s protocol. NanoDrop 2000 spectrophotometer (Thermo) and Bioanalyzer 2100 (Agilent) were used to determine the quality and quantity of total RNA.

Microarray assays were performed on Affymetrix GeneChip miRNA 4.0 Array, which contains 3164 mouse miRNA probe sets. The dataset was imported to Microsoft Excel. After normalizing the signal of each microRNA, the expression level was computed, and Student’s t-test was performed to estimate the between-group differences.

### Statistical analysis

Quantitative data are expressed as mean ± standard deviation (SD). Paired data were evaluated by Student’s t-test. Multiple comparisons were performed by one-way ANOVA with the Bonferroni post hoc test (SPSS 16.0). P-values < 0.05 were considered to be statistically significant.

## Additional Information

**How to cite this article:** Li, G. *et al*. Cardiac repair in a mouse model of acute myocardial infarction with trophoblast stem cells. *Sci. Rep.* **7**, 44376; doi: 10.1038/srep44376 (2017).

**Publisher's note:** Springer Nature remains neutral with regard to jurisdictional claims in published maps and institutional affiliations.

## Supplementary Material

Supplementary Information
