# Supplementary material for: Cardiac repair in a mouse model of acute myocardial infarction with trophoblast stem cells
Source: Sci Rep. 2017 Mar 15;7:44376. doi: 10.1038/srep44376 (PMC5353648; doi:10.1038/srep44376)
Supplement: Supplementary Information [file srep44376-s1.pdf]

Supplementary Information

Cardiac repair in a mouse model of acute myocardial infarction with trophoblast stem cells

Guannan Li[1,3], Jianzhou Chen[1,3], Xinlin Zhang[1], Guixin He[1,2] ,Wei Tan[2] ,Han Wu[1], Ran Li[1] ,Yuhan Chen[1] ,Rong Gu[1], Jun Xie[1,*] , Biao Xu[1,*]

[1]Department of Cardiology, Nanjing Drum Tower Hospital, the Affiliated Hospital of Nanjing University Medical School, Nanjing, Jiangsu, 210008, China.

[2]Department of Cardiology, the First Affiliated Hospital of Guangxi University of Chinese Medicine, Nanning, Guangxi, 530023, China.

[3]These authors contributed equally to this work.

[*]Correspondence: J.X. (darcy_pann@hotmail.com) or B.X. (xubiao@medmail.com.cn)

Supplemental Methods

Cell characterization by immunofluorescence

For characterization, stem cells were seeded on 24-wells plate coverslips, fixed with 4% buffered paraformaldehyde (PFA) for 20min at RT. TSCs was incubated with primary antibody of Cdx2 (1:200, Abcam), and MSCs was incubated with primary antibodies of CD90.2, CD105, CD31, CD45, CD117 (1:100, BD biosciences) for 16hrs at 4 ℃ (overnight), then cells were incubated with a labelled secondary antibody (1:500, Invitrogen) for 40 minutes at 37 ℃. Nuclei were recognized by 4', 6-diamidino-2-phenylindole (DAPI) staining. Incubations process were carried out in humid chambers in the dark. Cells were analyzed at a 40 × magnification.

Real-time PCR analysis of TSCs

Total RNA from TS cell lines (n=3) was extracted using TRIzol (Invitrogen). Then cDNA was produced by using PrimeScript RT reagent Kit (TaKaRa) and mRNA expression was analyzed with quantitative real-time RT-PCR using a SYBR Premix Ex Taq System (TaKaRa). Sequences of the primers for *Cdx2, Tfap2c, Fgfr2, Eomes, Oct4* are shown as follows: *Cdx2* forward 5'-CACCGTGGGCTGAGGTGCAG-3', reverse 5'-AGTGGCGCACGGAGCTAGGA-3'; *Tfap2c* forward 5'-TTGCTCCTACACGATCAGACT-3', reverse 5'-AGAAGACCTCACTGGGGTTCA-3'; *Fgfr2* forward 5'-AATCTCCCAACCAGAAGCGTA-3', reverse 5'-CTCCCCAATAAGCACTGTCCT-3'; *Eomes* forward 5'-CGGTGGCTTGTCACGCCTGT-3', reverse 5'-GGCCTCCCCACCCTGCCATA-3';

and *Oct4* forward 5'-TCTTTCCACCAGGCCCCCGGCTC-3', reverse 5'-TGCGGGCGGACATGGGGAGATCC-3'.

Flow cytometry analysis of MSCs

MSCs were stained for surface antigen expression using the following antibodies: anti-CD90.2, anti-CD105, anti-CD31, anti-CD45, anti-CD117 (BD biosciences). After cell detachment, the first incubation was performed for 60 min at 37 ℃, then cells were washed in PBS and incubated with a labelled secondary antibody (Invitrogen) for 60 minutes at room temperature (RT), in the dark. After staining, cells were washed in PBS and fluorescence was analyzed using a FACS Canto II flow cytometer and FACS Diva software (both BD Biosciences).

Echocardiographic Measurements

Cardiac function was evaluated by transthoracic echocardiography before (baseline) and at 14 and 21days after MI. The measurement was performed by an observer blinded to treatment. Studies were recorded with a dynamic focused 30-MHz probe echocardiography machine (Visual Sonics Vevo2100, Canada). Briefly, mice were anesthetized using isoflurane and transferred to an imaging stage equipped with a warming pad for controlled maintenance of mouse body temperature at 37 ℃ and a built-in electrocardiography system for continuous heart rate (HR) and respiratory rate monitoring. The M-mode measurements of LV dimensions were averaged from more than 3 cycles. The end-diastolic left ventricular inner diameter (LVID;d) and end-

systolic left ventricular inner diameter (LVID;s) were measured. Percentage of left ventricular fractional shortening (FS) was calculated as follows: %FS = (LVIDd-LVIDs)/LVIDd*100. Left ventricular ejection fractional (EF) was calculated as follows: %EF = ($LVIDd^2$-$LVIDs^2$)/$LVIDd^2$*100.

Tissue preparation

After the detection of echocardiography, the mice were etherized and sacrificed, hearts was arrested by injection of potassium chloride (10% 0.5ml). Once hearts were harvested, perfused with PBS, and then 8 hearts of each group were fixed with 4% paraformaldehyde, paraffin embedded, sectioned, and stained with the hematoxylin eosin (HE), Masson's trichrome and immunohistochemistry experiments. Six hearts of each group were frozen in liquid nitrogen and transferred to store at -80℃ for the microarray analysis and RT-PCR. Six hearts of TSCs group or MSCs group were embedded in OCT compound (Sakura), snap-frozen in liquid nitrogen, and then store at -80℃ for the detect of the retention and differentiation of stem cells after transplantation.

Histological Examination on paraffin-embedded tissue sections

Paraffin-embedded hearts were cut in 4-μm sections for the next staining. The measurement of infarct size, at the level of the mid-papillary heart muscles, was scored following HE staining and calculated as percentage of the whole LV with Image Pro Plus6.0 software (ipp6.0). The wall thickness of the border zone (WTBZ) of LV and

the thickness of infarcted myocardium (TIM) of the LV are schematically illustrated as previously described. Six sections per heart, from 8 hearts in each group, were analyzed and averaged.

The measurement of fibrosis were stained with Masson’s trichrome staining. Twelve images from the border zone of each heart were obtained and 8 hearts per group were included, and images were analyzed using ipp6.0. The collagen content was calculated as a percentage of the area of each image.

The measurement of capillary density was assessed by immunohistochemical staining with CD31 antibody (1:200, Abcam). Angiogenesis was measured by counting the number of CD31-positive cells per field in 6 randomly chosen microscopic fields from 6 different sections in either infarct zone or border zone with ipp6.0, and 8 hearts per group were included.

The measurement of apoptotic cells were evaluated by terminal deoxynucleotidyl transferase-mediated dUTP nick end labelling (Tunel) assay with an in situ cell death detection kit (Roche, POD). Hematoxylin was used for counterstaining. The percentage of TUNEL-positive cells in the border zone and infarct zone were compared to the total number of nuclei per field, six randomly fields from 6 different sections, and 8 hearts each group were measured using ipp6.0.

The measurement of cell proliferation was analyzed by immunohistochemical staining with Ki67 antibody (1:100, Abcam). The proliferation in either infarct zone or border zone was measured by counting the number of Ki67-positive cells per field within 6 randomly fields from 6 different sections using ipp6.0, and 8 hearts per group

were included.

Histological Examination on frozen tissue sections

The OCT-embedded frozen hearts was cut in 7-μm cryosections for the immunofluorescent staining. Retention of injected cells was detected by the percent of GFP^+ cells to all cells in every field under fluorescent microscopy, and nuclei were counterstained with DAPI. The fate of transplanted cells were performed the Tunel for apoptosis and phosphorylated Histone-H3 (pH3) staining for proliferation. The co-expression of GFP and Tunel indicated the apoptosis of implanted cells, and the co-expression of GFP and pH3 indicated the proliferation of implanted cells. Quantification of the apoptosis and proliferation of TSCs and MSCs was detected by the percent of $Tunel^+$ or $pH3^+$ cells to GFP^+ cells in every field under fluorescent microscopy.

Differentiation of stem cells was staining with α-actinin, CD31 and vWF, nuclei were counterstained with DAPI.

The proliferation of host cardiomyocytes in TSCs or MSCs groups was co-staining with α-actinin, pH3 and DAPI every cryosection.

Primary antibodies were used as follows: Tunel assay with an in situ cell death detection kit (Roche, TMR red); rabbit pH3 antibody (1:200, Abcam); mouse α-actinin antibody (1:200, Abcam); rabbit CD31 antibody (1:200, Abcam); rabbit vWF antibody (1:200, Abcam). Second antibodies were used as follows: donkey anti-mouse IgG Alexa-Fluor® 488 (1:500, Invitrogen); donkey anti-rabbit IgG Alexa-Fluor® 594 (1:500,

Invitrogen). Cells were counted in 6 HPFs per section, 6 sections per animal.

Real-time qPCR

Nine miRNAs (*miR-455-5p, miR-330-5p, miR-122-5p, miR-3058-5p, miR-6370, miR-7093-5p, miR-467h, miR-200b-3p, miR-375-3p*) were selected from the array data analysis for validation. TaqMan microRNA assays (Applied Biosystems) were used to quantify the expression of miRNAs. Amplication and detection were performed using 7500HT Fast Real-Time PCR system (Applied Biosystems). The miRNA levels were normalized to U6 snRNA as an internal control. Relative abundance of each miRNA was calculated by $2^{-\Delta\Delta Ct}$ method and the results were assessed using t-test.

The sequences of the primers for real-time qPCR are shown as follows: *mmu-miR-455-5p* UAUGUGCCUUUGGACUACAUCG; *mmu-miR-330-5p* UCUCUGGGCCUGUGUCUUAGGC; *mmu-miR-122-5p* UGGAGUGUGACAAUGGUGUUUG; *mmu-miR-3058-5p* UCAGCCACGGCUUACCUGGAAGA; *mmu-miR-6370* GCAGGAACAGCAAAGGGGAAG; *mmu-miR-7093-5p* CAGGAUGACAGAAGGAAAACCU; *mmu-miR-467h* AUAAGUGUGUGCAUGUAUAUGU; *mmu-miR-200b-3p* UAAUACUGCCUGGUAAUGAUGA; and *mmu-miR-375-3p* UUUGUUCGUUCGGCUCGCGUGA.

Supplementary Figures and Tables

Supplementary Figure S1.

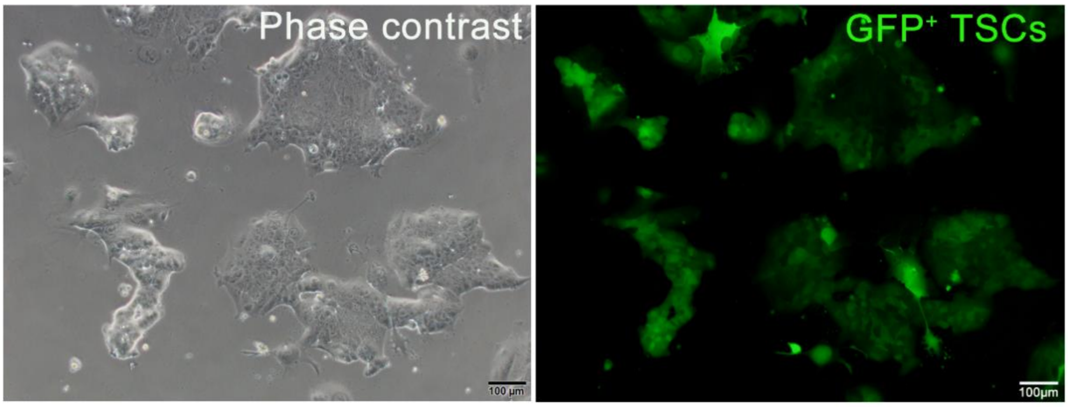

Figure S1: Characterization of TSCs. TSCs expressed GFP under fluorescent microscopy.

Supplementary Figure S2.

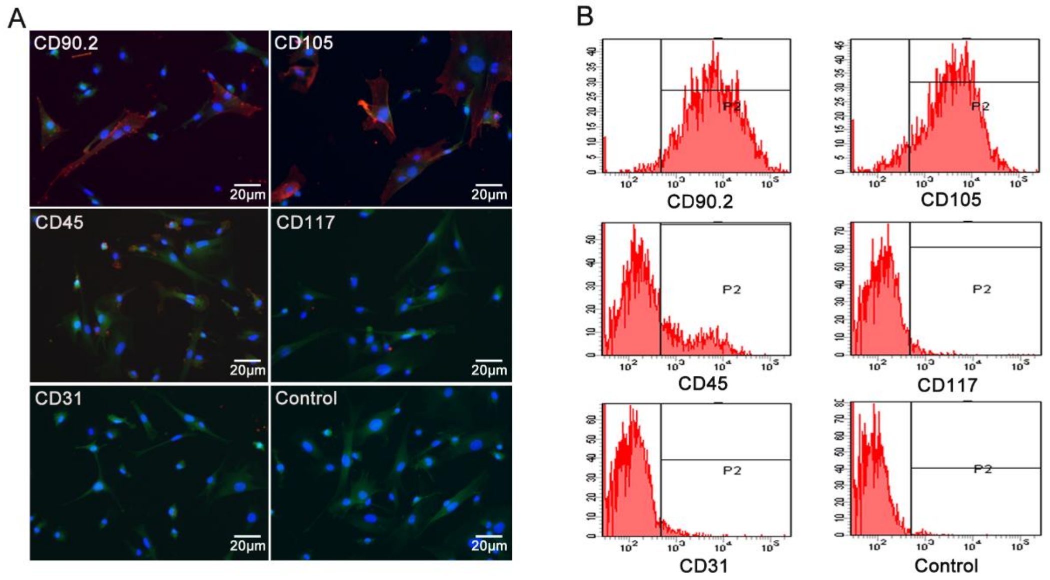

Figure S2: Characterization of TSCs. (A) MSCs expressed CD90.2 and CD105, and negative for CD31, CD45, and CD117 by immunofluorescent staining. (B) Flow cytometry analysis showed that MSCs expressed CD90.2(96.43 ±0.58%), CD105(91.46 ±0.79%), CD31(3.9 ±0.62%), CD45(20.87 ±2.13%), and CD117(1.37 ±0.36%). Data are depicted as mean ± SD.

Supplementary Figure S3.

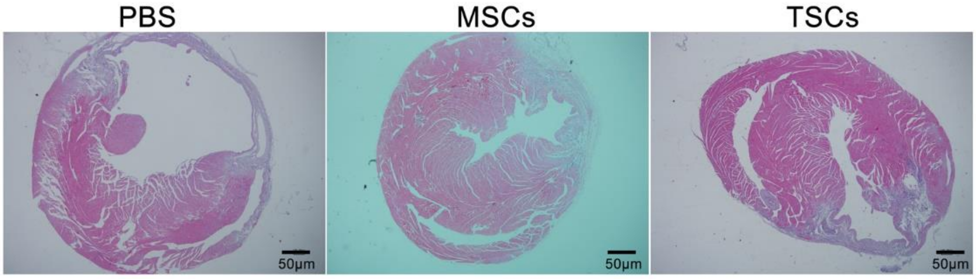

Figure S3: Representative HE-stained histological sections at 3 weeks after cell transplantation.

Supplementary Figure S4.

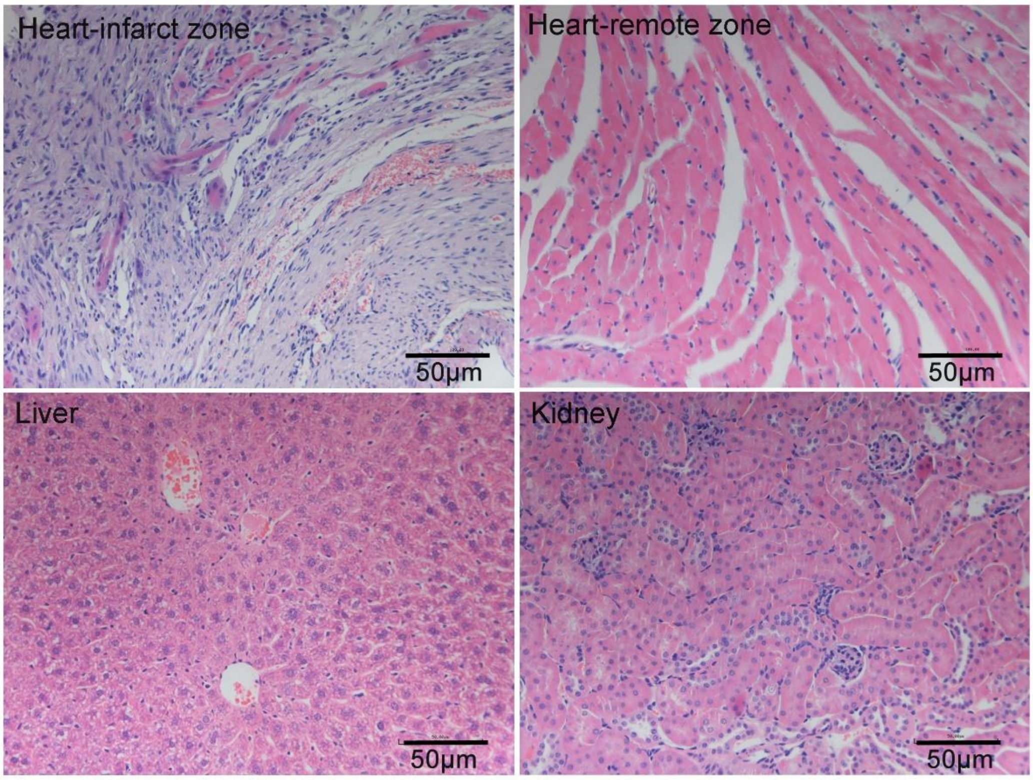

Figure S4: No tumor formation was observed in the major organs of mice transplanted with TSCs and MSCs 3 weeks after delivery.

Supplementary Figure S5.

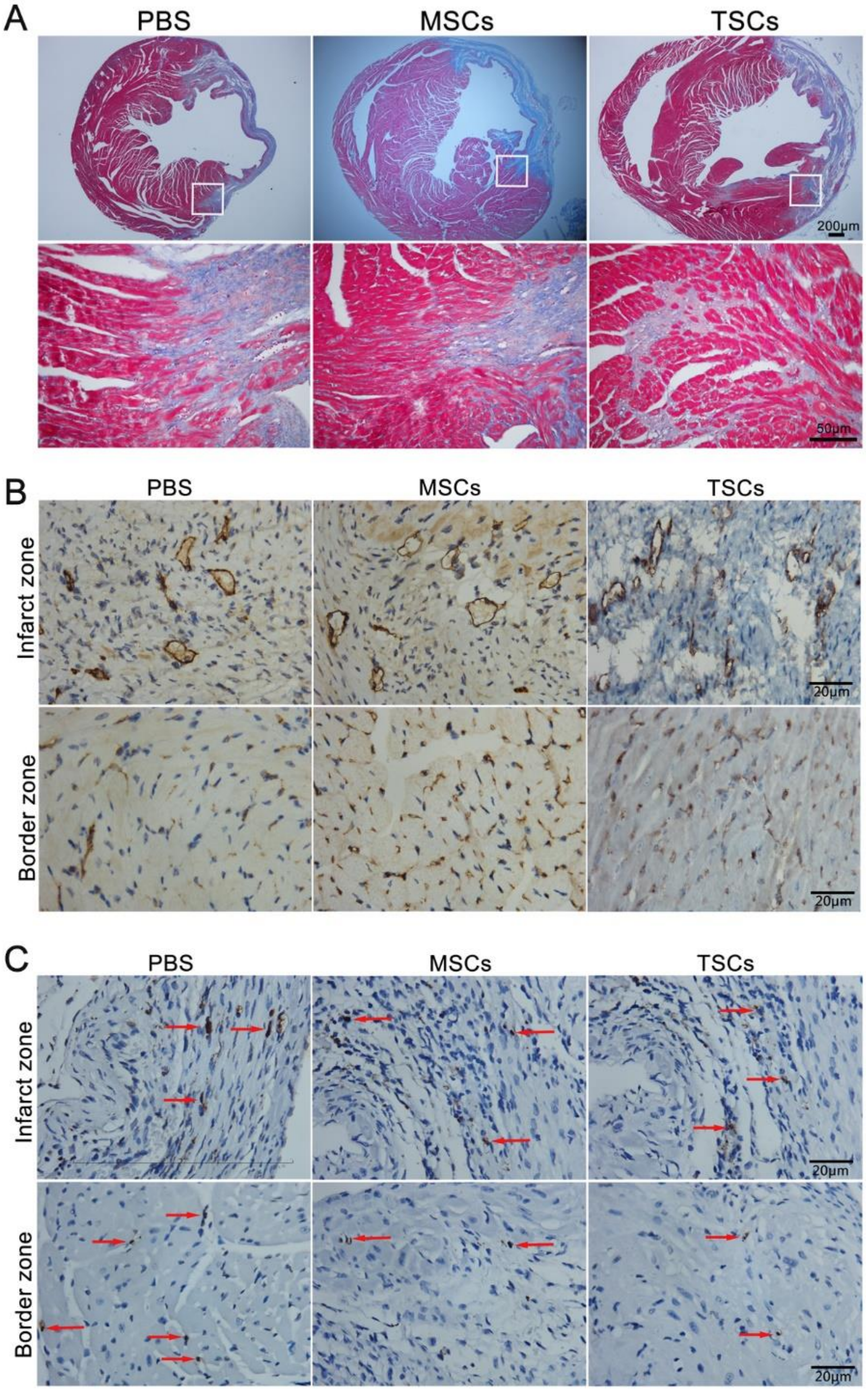

Figure S5: (A) Masson trichrome-stained sections from the hearts of three groups at 3 weeks after cell transplantation. (B) Representative CD31-stained histological sections in infarct zone and border zone of hearts at 3 weeks after cell transplantation. (C) Representative Tunel-stained histological sections in infarct zone and border zone of hearts at 3 weeks after cell transplantation.

Supplementary Table S1.

Quantification of the retention of TSCs and MSCs after transplantation. (*$p<0.05$)

	MSCs group			TSCs group		
**n**	**DAPI$^+$**	**GFP$^+$**	**GFP$^+$/DAPI$^+$**	**DAPI$^+$**	**GFP$^+$**	**GFP$^+$/DAPI$^+$**
**1**	8070	857	10.62%	7452	1314	17.63%
**2**	8016	956	11.93%	8320	1639	19.70%
**3**	7441	882	11.85%	9998	2052	20.52%
**4**	7896	905	11.46%	7804	1676	21.48%
**5**	6928	724	10.45%	7141	1408	19.72%
**6**	8492	1072	12.62%	6943	1290	18.58%
**Sum**	46843	5396	11.49 ±0.76%	47658	9379	19.60 ±1.25% *****

Supplementary Table S2.

Quantification of the proliferation of TSCs and MSCs after transplantation. (*$p<0.05$)

	MSCs group			TSCs group		
**n**	**GFP$^+$**	**pH3$^+$**	**pH3$^+$/GFP$^+$**	**GFP$^+$**	**pH3$^+$**	**pH3$^+$/GFP$^+$**
**1**	726	38	5.23%	815	68	8.34%
**2**	647	27	4.17%	922	79	8.57%
**3**	867	31	3.58%	1176	81	6.89%
**4**	534	24	4.49%	917	52	5.67%
**5**	501	22	4.39%	1082	85	7.86%
**6**	665	30	4.51%	837	77	9.20%
**Sum**	3940	172	4.40 ±0.49%	5749	442	7.75 ±1.17% *****

Supplementary Table S3.

Quantification of the apoptosis of TSCs and MSCs after transplantation.

	MSCs group			TSCs group		
**n**	**GFP$^+$**	**Tunel$^+$**	**Tunel$^+$/GFP$^+$**	**GFP$^+$**	**Tunel$^+$**	**Tunel$^+$/GFP$^+$**
**1**	625	46	7.36%	972	72	7.41%
**2**	540	39	7.22%	1042	67	6.43%
**3**	686	37	5.39%	1120	70	6.25%
**4**	592	41	6.93%	1075	60	5.58%
**5**	602	50	8.30%	870	49	5.63%
**6**	706	42	5.95%	993	52	5.24%
**Sum**	3751	255	6.86 ±0.95%	6072	370	6.09 ±0.72%

Supplementary Table S4.

Quantification of the proliferation of endogenous cardiomyocytes in MSCs and TSCs group. (*$p<0.05$)

	MSCs group			TSCs group		
**n**	**α-actinin$^+$**	**pH3$^+$**	**Percentage**	**α-actinin$^+$**	**pH3$^+$**	**Percentage**
**1**	2475	31	1.25%	2610	60	2.30%
**2**	2834	34	1.20%	2636	51	1.93%
**3**	2611	28	1.07%	2659	75	2.82%
**4**	2592	40	1.54%	2806	58	2.07%
**5**	2397	38	1.58%	2473	68	2.75%
**6**	2733	23	0.84%	2559	62	2.42%
**Sum**	15642	194	1.25±0.26%	15749	442	2.38±0.33% *****

Supplementary Table S5.

Up-regulated and down-regulated microRNAs in MSCs and TSCs treated hearts compared with PBS treated hearts.

MSCs vs PBS		TSCs vs PBS	
**miRNA ID**	**Fold Change**	**miRNA ID**	**Fold Change**
mmu-miR-455-5p	6.765337935	mmu-miR-19a-3p	2.368532279
mmu-miR-199a-5p	2.760500763	mmu-miR-1983	2.348159379
mmu-miR-199b-5p	2.613077657	mmu-miR-8119	2.3367607
mmu-miR-330-5p	2.555625902	mmu-miR-455-5p	2.171535192
mmu-miR-214-5p	2.385712176	mmu-miR-184-3p	2.171175982
mmu-miR-370-3p	2.372631419	mmu-miR-330-5p	2.158616066
mmu-miR-8119	2.291030503	mmu-miR-7216-5p	2.149493379
mmu-miR-7688-5p	2.2663814	mmu-mir-465c-1	2.102311816
mmu-miR-214-3p	2.228387302	mmu-mir-465c-2	2.102311816
mmu-miR-411-3p	2.210551992	mmu-miR-200b-3p	-3.981871507
mmu-miR-7079-5p	2.20221196	mmu-miR-375-3p	-3.211094968
mmu-miR-668-3p	2.083530183	mmu-miR-200c-3p	-2.919544565
mmu-miR-21a-5p	2.070773843	mmu-miR-3058-5p	-2.871782856
mmu-miR-199a-3p	2.020856201	mmu-miR-6241	-2.614424364
mmu-miR-199b-3p	2.020856201	mmu-miR-122-5p	-2.494383575
mmu-miR-122-5p	-13.32348879	mmu-miR-669e-5p	-2.356142288
mmu-miR-429-3p	-3.063512349	mmu-miR-200a-3p	-2.25994344
mmu-miR-3058-5p	-2.915015053	mmu-miR-124-3p	-2.226189905
mmu-miR-451a	-2.774895291	mmu-miR-193a-3p	-2.165069494
mmu-miR-200b-5p	-2.637997749	mmu-miR-669d-5p	-2.155282031
mmu-miR-200c-3p	-2.62480455	mmu-miR-669l-5p	-2.128105981
mmu-miR-3076-5p	-2.185484783	mmu-mir-124-3	-2.126975379
mmu-miR-6370	-2.163449332	mmu-mir-124-2	-2.126975379
mmu-miR-7093-5p	-2.069178826	mmu-mir-124-1	-2.126975379
mmu-miR-467h	-2.003450225	mmu-miR-145b	-2.106249971
		mmu-miR-466h-5p	-2.010698358

The positive number of fold change means up-regulated, and the negative number of fold change means down-regulated.

Supplementary Table S6.

Representative miRNAs (fold change > 2.0 and P-value < 0.05) confirmed by qRT-PCR.

MSCs vs PBS			TSCs vs PBS		
**miRNA ID**	**Fold Change**	**p-value**	**miRNA ID**	**Fold Change**	**p-value**
mmu-miR-455-5p	6.76534	0.02437	mmu-miR-455-5p	2.17154	0.01579
mmu-miR-330-5p	2.55563	0.00835	mmu-miR-330-5p	2.15862	0.03093
mmu-miR-122-5p	-13.32349	0.01261	mmu-miR-200b-3p	-3.98187	0.02776
mmu-miR-3058-5p	-2.91502	0.03409	mmu-miR-375-3p	-3.21109	0.03082
mmu-miR-6370	-2.16345	0.04666	mmu-miR-3058-5p	-2.87178	0.00490
mmu-miR-7093-5p	-2.06918	0.00383			
mmu-miR-467h	-2.00345	0.04097			

The positive number of fold change means up-regulated, and the negative number of fold change means down-regulated.
